# Effect of hand hygiene intervention on the absenteeism of pre-school children in Klang Valley, Malaysia: a quasi-experimental study

**DOI:** 10.1007/s12519-019-00283-x

**Published:** 2019-07-08

**Authors:** Nurul Azmawati Mohamed, Mohd Dzulkhairi Mohd Rani, Tengku Zetty Maztura Tengku Jamaluddin, Zarini Ismail, Shalinawati Ramli, Habibah Faroque, Farisha Nur Abd Samad, Abdul Rashid Ariffien, Aisyah Ar Redha Che Amir Farid, Ilina Isahak

**Affiliations:** 1 Faculty of Medicine and Health Sciences, Universiti Sains Islam Malaysia, Pandan Indah, 55100 Kuala Lumpur, Malaysia; 2 Faculty of Medicine and Health Sciences, Universiti Putra Malaysia, 43400 Serdang, Selangor Malaysia

**Keywords:** Absenteeism, Hand hygiene, Povidone-iodine, Pre-school children

## Abstract

**Background:**

Absenteeism amongst pre-school children is often due to illnesses such as hand, foot, and mouth disease, acute gastroenteritis, cold and flu, which are easily spread amongst them. This is because of weak immunity and lack of knowledge on proper hand hygiene. This quasi-experimental study assessed the efficacy of an intervention consisting of a hand hygiene education programme, along with digital tools in bringing about a change in behaviour and health conditions amongst pre-school children in Klang Valley, Malaysia.

**Methods:**

A total of 377 school children, male and female, aged 5–6 years old, participated and were assigned to either the intervention or a control group. During the 2 months intervention period, children in the test group were trained on proper hand hygiene practices and techniques with the aid of the interactive android-based tablets. The numbers of absent days of all the children were recorded for 2 months before the intervention and during the intervention.

**Results:**

In the test group, there was a 25% increase in the total number of absent days from the pre-intervention period to the intervention period, a much lesser increment observed as compared to that of control group in which the increase was much higher at 89%. Results showed a significant difference (*P* < 0·05) between the absenteeism rates for the test and control group during the intervention period.

**Conclusion:**

These results suggest that proper education and intervention increase hand hygiene compliance, which may help decrease school absenteeism due to illness; however, a longer study duration may be necessary to evaluate the benefit further.

## Introduction

Absenteeism is an important education issue, as students who are frequently absent may fall behind on the curriculum [1]. A majority of school absences can be attributed to illness, accounting for up to 75% of absences [2]. Children in the pre-school age group are more susceptible to gastrointestinal and respiratory infections [3]. These diseases are easily transmitted between young children at pre-school because of their naturally weak immune systems and the large amount of time they spend in close proximity to each other [4], in addition to the lack of knowledge on basic hygiene practices and habits [3] such as hand washing.

It has been deduced in several other studies that infectious diseases can be prevented by performing proper hand washing using an antiseptic hand cleanser or hand rub [2, 5, 6]. Not only is improving hand washing behaviour effective in preventing disease transmission, it is also more economical compared to other improvements to the hygiene infrastructure [7]. Therefore, it is believed that by introducing a comprehensive hygiene awareness programme at the pre-school level, the disease burden and the absenteeism rate can be lowered, subsequently improving academic performance [1].

Different approaches of intervention have already been investigated in previous reported studies such as the direct contact hand washing intervention [8], different time interval applications of alcohol hand gel [9] and, multi-level interventions [1]. All these methods were reported to be effective in reducing the incidence of infectious illnesses. These interventions are more likely to be effective in pre-school age children, as their hygiene habits have not yet been established and are therefore easier to be modified. Additionally, cultivating good hygiene practices at a younger age can have a long-lasting impact on their health [10].

This study was conducted to assess the efficacy of a comprehensive hand hygiene intervention consisting of a hand hygiene education programme, with electronic monitoring of hand washing steps in real time, in lowering absenteeism rate.

## Methods

### Study design and place of study

This quasi-experimental study was done from August to November 2017. Pre-schoolers under the management of Majlis Agama Islam Wilayah Persekutuan (MAIWP), Malaysia were selected, because the pre-schools cater exclusively to children aged five and six. After obtaining the permission letter from MAIWP to their schools, the potential pre-schools were approached to invite them to participate in the hand hygiene programme. The pre-schools were selected using Excel Spreadsheet Randomisation Command by the simple random sampling method. School P (*n* = 297) was assigned as the test group and School C (*n* = 193) was assigned as the control group.

Parents were informed of the study through the information sheets provided, and consent was obtained through the attached form. The information sheet clearly mentioned about exclusion from the study if a child has allergies towards povidone-iodine-containing products and/or a thyroid disorder. None of the parents reported allergy or a thyroid disorder.

### Study plan

The sample size required was calculated using 52% for prevalence of children with adequate knowledge on hygiene, 99% confidence interval (CI) and, the power of study 95% [11]. An online sample size calculator, Open Epi was used. To cover for non-response and dropouts, 30% was added to the sample size. Therefore, the number of participants required for this study group was 138. This study involved two phases: pre-intervention and intervention phases.

Throughout the pre-intervention period (August and September), absenteeism forms were distributed to each class teacher in which they had to fill in the dates, name of absentees, the number of days absent and, the reasons for being absent. The intervention phase for the test group commenced in early October. The test group was assigned to receive: (1) a hand hygiene slot including 4 hours educational session on hand hygiene importance and physical demonstration as well as practices of correct hand washing steps (based on the World Health Organization recommendation) with jingle on the first day of intervention period; (2) educational materials and posters for display at a common area, corridor and at the basins; (3) proper hand washing facilities including 7.5% povidone-iodine skin cleanser as the standard hand washing agent, paper towels and waste bins; (4) installation of electronic devices with interactive hand hygiene application at the washing basins to monitor and encourage proper hand washing steps in real time.

Throughout the study, the hand washing practices were guided by the application on the tablets and posters. Attendance forms similar to the ones given in the pre-intervention period were distributed to the teachers to be filled for the month of October and November. For the control group, similar absenteeism forms were distributed to class teachers for the months of August and September (pre-intervention period) and for the months of October and November. Children in the control group were asked to follow their usual hand hygiene practices that include washing hands before and after eating.

### Data analysis

Demographic information collected is presented descriptively, whereas the absenteeism rates were analysed analytically using IBM Statistical Package for Social Sciences (SPSS) Version 22. The average number of absent days was calculated for both the pre-intervention months (August, September) and post-intervention months (October, November) and compared by analysis of variance (ANOVA**)** and Student’s *t* test**.** A *P* value of less than 0.05 (*P* < 0.05) was considered to be statistically significant.

## Results

Data from classes that did not submit the attendance form for any of the 4 months of study or with incomplete attendance forms, and students who did not obtain consent from their parents were excluded from analysis, leaving a final sample of 184 children for the test group and 193 children in the control group. Table 1 illustrates the demographic characteristics of the study participants.

**Table 1:** Demographic characteristics of participants

Variables	Test group		Control group	
	*n*	%	*n*	%
Male	52	28.3	93	48.2
Female	132	71.7	100	51.8

The percentage of absent days, which is calculated as the ratio of total absent days to total possible days of attendance for each month is shown in Table 2. The total possible days of attendance is obtained by multiplying the number of school days for that period with the total number of students. In the test group, there was a 25% increase in the total number of absent days from the pre-intervention period to the post-intervention period, while in the control group, the increase was much higher at 89%.

**Table 2:** Total rate of absenteeism during the period of study

Variables	Test group			Control group		
	Total number of absent days	Total number of possible days of attendance	% total number of absent days	Total number of absent days	Total number of possible days of attendance	% total number of absent days
Pre-intervention						
August	262	3496	7.5	333	3667	9.1
September	242	3496	6.9	225	3667	6.1
Total	504	6992	7.2	558	7334	7.6
During intervention						
October	224	2944	7.6	271	3088	8.7
November	273	2576	10.6	564	2702	20.8
Total	497	5520	9.0	835	5790	14.4

The average absent days (number of days absent per student) stated in the table above according to the phase of study. A higher average was seen in the pre-intervention period for the test group, whereas the opposite was seen in the control group. Analysis of variance (ANOVA) revealed no significant difference between the average number of absent days for the test group within the 4 months of study (*P* > 0.05) as shown in Table 3. However, there is significant difference between the average numbers of absent days for the control group within the 4 months of study (*P* > 0.05). Post hoc analysis shows that the average number of absent days for the month of November was significantly higher than that of all the other months.

**Table 3:** Analysis of variance (ANOVA) comparison of the average number of absent days for the test group

Variables	Sum of squares	*df*	Mean square	*F*	Sig.
Between groups	29.593	3	9.864	1.069	0.362
Within groups	2997.811	325	9.224		
Total	3027.404	328			

Student’s *t* test for the test group revealed no significant difference (*P* > 0.05) between the average number of absent days in the pre-intervention period and during the intervention period. When comparing between the two groups, Table 4 shows that during the intervention phase, the average number of absent days was significantly lower in the test group.

**Table 4:** Student’s* t* test between the test and control groups during the intervention period

Variables	Levene's test for equality of variances		Student’s *t* test for equality of means		
	*F*	Sig	*t*	*df*	Sig. (two-tailed)
Number of days absent					
Equal variances assumed	26.553	0.000	4.801	442.000	0.000
Equal variances not assumed			4.819	377.936	0.000

## Discussion

Absenteeism is a serious educational issue and appropriate measures need to be explored to prevent further deterioration. This is because absenteeism is one of the reasons for the decline in the academic performance of school children [1]. Absentees might be left behind in their syllabus once they become absent from school. Especially in pre-school, this can have a significant impact as education in early childhood focusing on 5- and 6-year-old children is crucial, as it serves as their preparation to enter the primary education level [12].

Based on the results obtained for the control group, by comparing the number of days absent in the pre-intervention phase with the other 3 months, it was shown that there is a significant difference between these months where November was recorded with the highest average number of days absent. Moreover, the results obtained for the test group in this study shows no significant difference between the absenteeism rates in the pre-intervention and intervention phases, suggesting that the introduction of the hand hygiene intervention might have a protective effect on the target outcome on this group. The usage of antiseptic skin cleanser throughout the study period in the test group would contribute to maintaining good health among the children. This has been strongly supported by several other outcomes in different studies involving the povidone-iodine (PVP-I)-contained antiseptics. In these studies, it was proven that PVP-I is effective in eliminating a broad spectrum of virus and bacteria counts which are involved in causing infectious diseases such as common cold, influenza, oropharyngeal disorders and, hand, foot, and mouth disease [13–17].

Based on the results obtained from both groups, the average number of absent days is significantly higher in the post-intervention period (Table 5). However, when compared between both groups, the absenteeism rate is significantly lower in the test group during the post-intervention period as compared to the control group. Therefore, this has proven that this comprehensive hand hygiene module (intervention), antiseptic skin cleanser, BETHADINE^®^ (a registered trademark of Mundipharma) with the aid of hand washing application tablet introduced in the test group in this study was quite effective in reducing the rate of absenteeism and preventing infectious diseases among the pre-school children. This is in line with findings of previous studies carried out that have proven that absenteeism rates can be improved after introducing strict hand hygiene instructions and a comprehensive hand washing programme [2, 18]. Moreover, other hygiene practices such as cleaning the toilets, floors, furniture and, toys are also important to prevent disease transmission [19]. One of the reasons that can explain the apparent lack of effectiveness is that person-to-person transmission is not the only mode of disease transmission that exists in a pre-school. This is also supported by Nesti and Goldbaum where routine cleaning of toys and surfaces in the pre-school is recommended as a measure to reduce the spread of infection [3].

**Table 5:** Average number of absent days for those who were absent for at least 1 day

Average number of absent days	Test group		Control group	
	August and September	October and November	August and September	October and November
Total	3.09	2.99	2.54	3.73

Besides that, rather than focusing on just the students in teaching hand hygiene, involvement of teachers and parents also play an important role in sustaining a social standard of hand washing and, knowledge on hand hygiene among these young children [20]. Including the teachers or caretakers in the intervention is paramount in infection control [3]. This hand hygiene programme was mostly aimed at the students with the video and song material composed to suit their demographic, with minimal material to encourage proper hand hygiene practice amongst the teachers and parents. Therefore, further improvements can be made to the module to engage the caretakers as well, so they are able to pass on the knowledge to the children under their care. Several similar studies conducted to address on the absenteeism issues among the pre-school children have been reported as a result of illnesses such as common cold, gastrointestinal or minor respiratory infections.

It has been reported that different time intervals of application of the alcohol hand gel could also significantly reduce the incidence of illness among pre-school children [9] in addition to providing the training, funding and, policy making during the intervention period [1]. This indicates that knowledge of proper hand washing techniques alone is not sufficient to curb illnesses, but knowing when to wash hands and, having adequate facilities to do so also influences the outcome of the intervention. This hand hygiene module can be further improved not only by providing training for the teachers but also encouraging them to ensure that the knowledge is being put into practice by the students. In addition to that, the module must also emphasize the recommended times where hand washing should be done, such as after going to the washroom and before and after meals.

There are several strengths of this study that can be noted. This is the first study of its kind being conducted in Malaysia. Likewise, the school was provided with hand hygiene materials such as the antiseptic cleanser, tissue paper and, wastebaskets as part of the intervention module, thus providing a more encouraging environment for the students to improve their hand hygiene practice. Several studies have shown that accessibility to clean facilities and hand drying materials greatly influences hand hygiene compliance [21–24]. Additionally, this study targeted children aged five and six, during which their hygiene habits are still easily molded. Children can be easily encouraged and motivated to perform frequent hand washing especially before having meals and after defecation. It can be done by teaching them using fun hand washing songs or stories besides rewarding them based on their performance and techniques of hand washing [8]. For that reason, this study utilized video and song as part of the knowledge delivery process, along with interactive animations with tablet computers to instruct the children on the proper hand washing technique.

One drawback of this study was that the study was only done for a 4-month duration, towards the end of the school year. It is recommended that such a study should be implemented in the beginning of the school year to allow for the module to be implemented for a longer interval of time. The study can also be done during peak disease seasons, such as that done in Lau et al. to maximize the effect [2]. This follows the suggestion of Zomer et al. that the effectiveness of intervention is proportional to the increase in infections [19]. Long-term monitoring is an important component in promoting hand hygiene [25], as it can augment the awareness on hand hygiene and subsequently improve the effectiveness [26]. In other studies, the hand hygiene programmes were implemented for 6 months [19] and up to 8 months [2] before the effects were seen, with regular revision sessions organized in between. In this study, however, the module was introduced as a half-day event with reliance on tablet computers and posters to sustain the knowledge.

In conclusion, hand hygiene interventions have been shown to have a positive effect on infection rates and subsequently reduce absenteeism rates in pre-schools. However, the intervention efforts need to be complemented by the appropriate facilities and implementation at the appropriate time. Participation of teachers and parents is also crucial to make the intervention a success.
